# Simultaneous static and time-resolved nonenhanced peripheral MR angiography

**DOI:** 10.1186/1532-429X-15-S1-O60

**Published:** 2013-01-30

**Authors:** Ioannis Koktzoglou, Shivraman Giri, Parag Amin, Eugene Dunkle, Robert R Edelman

**Affiliations:** 1 Radiology, Northshore University Health System, Evanston, IL, USA; 2 The University of Chicago Pritzker School of Medicine, Chicago, IL, USA; 3 Northwestern University Feinberg School of Medicine, Chicago, IL, USA; 4 Siemens Healthcare, Chicago, IL, USA

## Background

Due to the association of gadolinium with nephrogenic systemic fibrosis in patients with renal insufficiency, nonenhanced methods for peripheral angiography such as quiescent interval single shot (QISS) and subtractive fast spin-echo are of increasing clinical interest. Despite the ability of these methods to depict peripheral vascular pathology, they have limited ability to display the arterial pulse wave which may aid in characterization of peripheral vascular disease. We describe a rapid nonenhanced MRA method that provides for static and time-resolved display of blood flow within the peripheral vasculature.

## Methods

This study was IRB approved. Imaging of 6 healthy subjects and 2 patients was performed on a 1.5T MRI system (MAGNETOM Avanto, Siemens Healthcare) with a cardiac-gated saturation-recovery 2D radial trueFISP sequence that acquired views over the entire cardiac cycle. A golden azimuthal angle increment (111.25°) was used to permit sliding window reconstruction at arbitrary temporal resolutions. The sequence was used to image the arteries of the lower and upper limbs. Typical imaging parameters were: TR/TE/flip of 4.1-4.5 ms/2.0-2.2 ms/90°, slice thickness 2.3-3.0 mm, in-plane spatial resolution 1.0-1.2 mm, 160 (upper limbs) to 432 (lower limbs) slices, 1 heartbeat/slice, 20 temporal frames/heartbeat, phase-based fat suppression, tracking saturation for venous suppression. A static angiogram was obtained by reconstructing all acquired views. A multi-shot implementation (2-4 heartbeats/slice) was tested for the purpose of improving temporal resolution.

## Results

**Figure 1:**
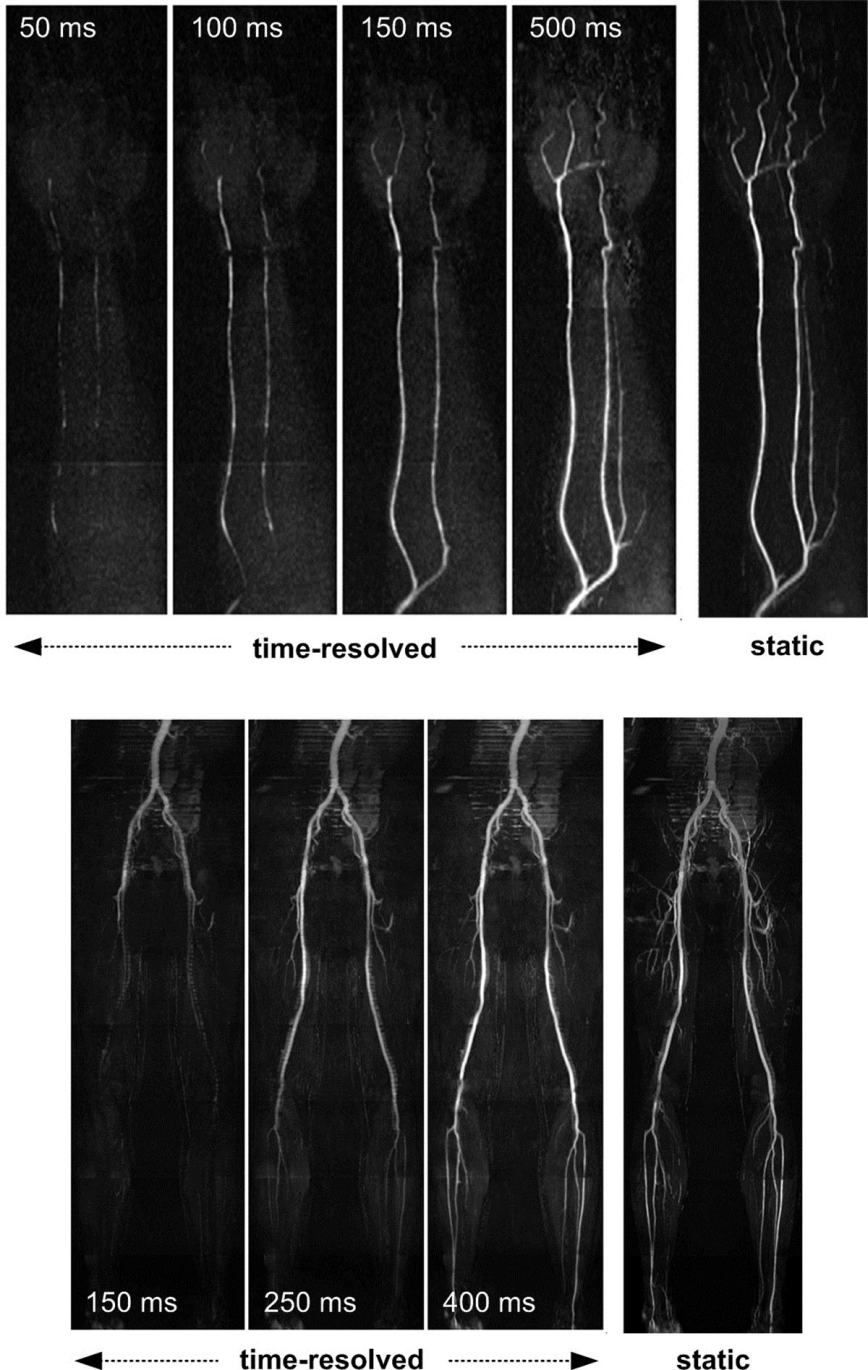
Static and select (3 or 4 of 20) time-resolved nonenhanced MR angiograms obtained with the proposed method in the upper (upper panel) and lower (lower panel) limbs. The propagation of flow from proximal to distal arterial segments is seen in the time-resolved angiograms; the static angiogram provides excellent display of arterial anatomy. Delay times relative to the electrocardiographic trigger are noted.

The described time-resolved nonenhanced MRA method depicted the onset and propagation of arterial flow in the peripheral arteries and provided static MR angiograms with excellent arterial conspicuity (Figure 1). Multi-shot imaging enabled the use of narrower data reconstruction window widths which improved the temporal resolution of the time-resolved series, and increased the signal to noise ratio of the static MR angiogram.

## Conclusions

Simultaneous static and time-resolved nonenhanced MRA of the peripheral arteries is feasible using the presented multi-slice 2D golden angle radial trueFISP method. The technique provides detailed three-dimensional angiograms of blood flow in the peripheral arteries and a static three-dimensional high spatial resolution MR angiogram. A multi-shot implementation allows the user to improve signal to noise ratio and temporal resolution.

## Funding

AHA SDG0835367N, NIH 1R01HL096916.

